# Interim Clinical Treatment Considerations for Severe Manifestations of Mpox — United States, February 2023

**DOI:** 10.15585/mmwr.mm7209a4

**Published:** 2023-03-03

**Authors:** Agam K. Rao, Caroline A. Schrodt, Faisal S. Minhaj, Michelle A. Waltenburg, Shama Cash-Goldwasser, Yon Yu, Brett W. Petersen, Christina Hutson, Inger K. Damon

**Affiliations:** ^1^Poxvirus and Rabies Branch, Division of High Consequence Pathogens and Pathology, National Center for Emerging and Zoonotic Infectious Diseases, CDC; ^2^Epidemic Intelligence Service, CDC; ^3^Office of the Director, Division of High Consequence Pathogens and Pathology, National Center for Emerging and Zoonotic Infectious Diseases, CDC; ^4^Regulatory Affairs, Division of Preparedness and Emerging Infections, National Center for Emerging and Zoonotic Infectious Diseases, CDC.

Monkeypox (mpox) is a disease caused by infection with *Monkeypox virus* (MPXV), an *Orthopoxvirus *(OPXV) in the same genus as *Variola virus*, which causes smallpox. During 2022, a global outbreak involving mpox clade IIb was recognized, primarily among gay, bisexual, and other men who have sex with men.* Most affected patients have been immunocompetent and experienced ≤10 rash lesions (*1*). CDC has recommended supportive care including pain control.^†^ However, some patients have experienced severe mpox manifestations, including ocular lesions, neurologic complications, myopericarditis, complications associated with mucosal (oral, rectal, genital, and urethral) lesions, and uncontrolled viral spread due to moderate or severe immunocompromise, particularly advanced HIV infection (*2*). Therapeutic medical countermeasures (MCMs) are Food and Drug Administration (FDA)–regulated drugs and biologics that are predominantly stockpiled by the U.S. government; MCMs developed for smallpox preparedness or shown to be effective against other OPXVs (i.e., tecovirimat, brincidofovir, cidofovir, trifluridine ophthalmic solution, and vaccinia immune globulin intravenous [VIGIV]) have been used to treat severe mpox. During May 2022−January 2023, CDC provided more than 250 U.S. mpox consultations. This report synthesizes data from animal models, MCM use for human cases of related OPXV, unpublished data, input from clinician experts, and experience during consultations (including follow-up) to provide interim clinical treatment considerations. Randomized controlled trials and other carefully controlled research studies are needed to evaluate the effectiveness of MCMs for treating human mpox. Until data gaps are filled, the information presented in this report represents the best available information concerning the effective use of MCMs and should be used to guide decisions about MCM use for mpox patients.

During May 2022, a global mpox outbreak was identified. A CDC clinical team began providing consultations^§^ to U.S. clinicians caring for patients with mpox, developing guidance and other online clinical resources for health care providers, and issuing health alerts^¶^ when emerging clinical concerns (e.g., severe infections in patients with advanced HIV infection) were detected. Before the 2022 outbreak, CDC experts in poxviruses and associated MCMs had evaluated efficacy data from animal models and reports of MCM use for a few human cases of related OPXV infections (e.g., vaccinia virus and cowpox virus). This information and unpublished data shared by intergovernmental partners guided initial clinical consultations; as more knowledge was acquired through clinical consultations, many of which involved repeated consultations and regular follow-up, CDC’s approach to mpox cases was refined. Recurrent questions that would benefit from expert input were identified (e.g., management of ocular infections); input was solicited from external experts in infectious diseases (including HIV), immunology, neurology, ophthalmology, dermatology, and public health emergency response. Identified experts included leaders of professional societies and physicians experienced in treating mpox during the current outbreak. Partners from the Public Health Emergency Medical Countermeasures Enterprise,** a U.S. intragovernmental committee that optimizes preparedness for public health emergencies (e.g., through developing and stockpiling available MCMs), were also consulted. This report is a comprehensive synthesis of the compiled evidence and is intended to foster strategic decision-making rather than serve as a prescriptive treatment guideline. Clinical considerations were developed in the context of limited data about MCM effectiveness during the current outbreak, finite supplies of some MCMs (e.g., VIGIV and intravenous [IV] tecovirimat), and a need to incorporate evolving data and clinical observations into guidance that can be used to manage cases, including in future months if case counts increase. The rationale for specific guidance is included.

## MCMs Being Used to Treat Mpox and Indications for Use

MCMs for OPXV infections include antivirals (i.e., tecovirimat, brincidofovir, cidofovir, and trifluridine ophthalmic solution) and VIGIV. Tecovirimat, brincidofovir, and VIGIV are recommended based on efficacy data from experimental animal models involving exposure to diverse OPXVs (i.e., variola, mpox, vaccinia, ectromelia, and rabbitpox viruses), albeit via the respiratory route, which is different from the close skin and mucosal contact associated with the ongoing mpox outbreak. Cidofovir and trifluridine ophthalmic solution are recommended because of their successful use treating other viral infections; cidofovir is also recommended based on data on the effectiveness of brincidofovir. All four antivirals were sporadically used to treat severe manifestations of human OPXV infections before the 2022 global outbreak (*3*–*7*); VIGIV has been used to treat adverse events from live, replicating vaccinia virus vaccines that are licensed to prevent smallpox (e.g., progressive vaccinia after receipt of Dryvax^§§^ or ACAM2000^¶¶^), and was used to treat smallpox disease before its 1980 worldwide eradication (*8*–*11*). Despite this real-world use, it is not known how often MCMs were associated with favorable outcomes and whether clinical improvements were due to MCMs, natural resolution of illness, or a combination of these.

MCMs have been widely used during the 2022 outbreak. As of February 2023, tecovirimat and VIGIV continue to be available through CDC-sponsored expanded access Investigational New Drug (IND) protocols; brincidofovir through an FDA–authorized single-patient emergency use IND; and cidofovir and trifluridine, commercially. To date, no data have shown whether MCMs are beneficial, including for pain control (irrespective of severity). Most persons recover with supportive care alone (including pain control***). MCMs (particularly tecovorimat) used without close monitoring could result in suboptimal drug levels and promote drug resistance,^†††^ thereby reducing its effectiveness for the individual patient and others. In addition, the effectiveness of MCMs for the treatment of mpox has not been systematically evaluated. For these reasons, CDC strongly encourages enrollment in clinical trials (e.g., the National Institutes of Health (NIH)–funded Study of Tecovirimat for Human Mpox [STOMP] trial).^§§§^

Severe mpox might manifest as hemorrhagic disease, many confluent or necrotic lesions, severe necrotizing or obstructive lymphadenopathy (e.g., of the upper airway), obstructive edema (e.g., of the gastrointestinal tract), extradermatologic manifestation (e.g., pulmonary nodules, encephalitis, myopericarditis, and ocular infections), and sepsis (*12*). Some patients might not have severe mpox at the time of first health care interaction but are at risk for severe mpox because of underlying medical condition (e.g., severe or moderate immunocompromise)^¶¶¶^ or presence of lesions on certain surfaces (e.g., penile foreskin, urethral meatus, or vulva). These might predispose patients to complications such as strictures or edema which could require procedures including urethral catheterization, colostomy, or surgical debridement. MCMs should be considered in these cases irrespective of patient immune status. Children and adolescents aged <18 years and pregnant persons have accounted for a small percentage (<0.3%) of total U.S. cases during the current outbreak, and when affected, have experienced mild illness (*13*,*14*); however, because these populations (particularly children aged <8 years) have historically experienced more severe clade I mpox infections, and because outcomes in pregnant women and neonates during the current outbreak might not be known for several months, case-by-case consideration of MCMs should be undertaken after weighing the potential benefits and harms.**** Other populations might also benefit from case-by-case consideration of MCM use. Persons with a history of atopic dermatitis and eczema (both well-controlled and not) might experience uncontrolled viral spread, possibly as a result of associated defects in the innate or adaptive immune response (*15*). Persons with extensive breaks in the dermal barrier (e.g., from burns, impetigo, varicella zoster virus infections, herpes simplex virus infection, severe acne, severe diaper dermatitis with extensive denuded skin, psoriasis, and Darier disease [keratosis follicularis]) might also be at risk for severe manifestations of uncontrolled viral spread depending on the severity of the underlying condition (*16*).

## Approach to Using MCMs to Treat Mpox

Through iterative consultations, a management algorithm outlining the approach to patients with suspected, probable, or confirmed mpox has been developed to aid in decision-making regarding the earliest use of effective MCMs when indicated (Figure). Coinfections (e.g., with syphilis, herpes simplex, varicella zoster, or molluscum contagiosum) should be considered. All patients with suspected mpox should be evaluated for preexisting immunocompromising conditions and be tested for HIV. No antiviral MCMs for use against OPXVs are virucidal, and optimal immune function is essential to recovery, irrespective of whether multiple MCMs are administered. Antiviral MCMs might complement the immune response by reducing replication, maturation, or spread of OPXVs. VIGIV might provide some level of passive immunity to certain patients with moderate or severe immunocompromise until a patient’s immune system is able to clear the virus. However, earliest optimization of immune function (e.g., by temporarily delaying or decreasing doses of chemotherapy and immunomodulatory therapies and by promptly initiating effective antiretroviral medications [ARVs] for treatment of HIV) is critical to favorable outcomes. Since August 2022, consultations with CDC have involved a large proportion of immunocompromised persons, particularly those with HIV and low CD4 cell counts (*12*). Comprehensive information about each MCM, including mechanism of action, safety, efficacy, and dosing should be reviewed along with the management algorithm when deciding about administration or cessation of MCMs (Table). Interactions with other medications including ARVs should also be considered (*17*).

**FIGURE:**
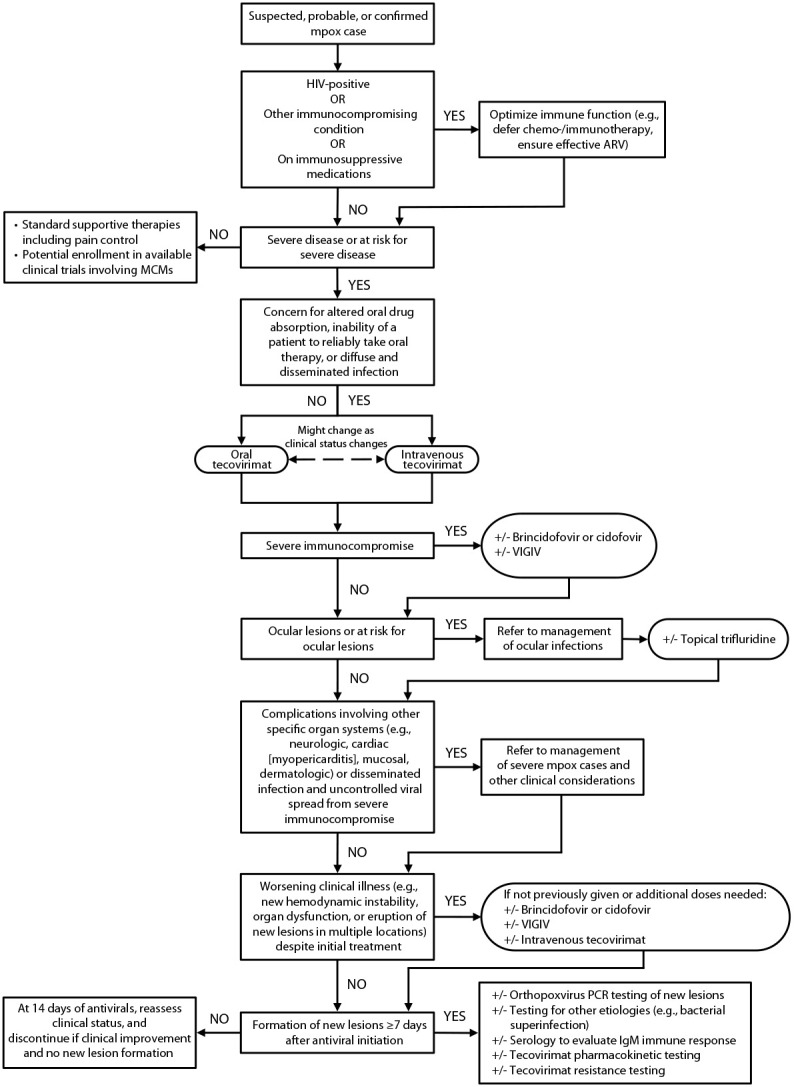
Approach to treatment^*^^,^^†^^,^^§^ of patients with severe^¶^ or at risk** for severe manifestations of mpox^††^ — United States, February 2023^§§^ **Abbreviations:** ARV = antiretroviral medications; GI = gastrointestinal; IgM = immunoglobulin M; MCM = medical countermeasure; PCR = polymerase chain reaction; VIGIV = vaccinia immune globulin intravenous. * Treatment includes MCMs (i.e., tecovirimat, brincidofovir, cidofovir, VIGIV, and trifluridine) and supportive therapies, including pain management. https://www.cdc.gov/poxvirus/monkeypox/clinicians/pain-management.html ^†^ Most immunocompetent patients should display signs of clinical improvement within 4 days of antiviral initiation (i.e., tecovirimat, brincidofovir, cidofovir, and trifluridine). Tecovirimat is expected to reach steady state concentrations by day 6 of dosing in healthy volunteers; therefore, worsening clinical illness after 7 days of treatment in patients with severe illness could prompt additional evaluations. ^§^ Concern for altered drug absorption includes the inability to tolerate or take oral therapy (e.g., nothing by mouth), or possibility that the oral drug absorption might be altered because of inability to consume a high-fat meal, severity of symptoms (e.g., systemic illness), comorbidities (e.g., history of gastric bypass or underlying GI disease), or other factors that might alter oral drug absorption. ^¶^ Hemorrhagic disease, a large number of confluent or necrotic lesions, severe lymphadenopathy that is necrotizing or obstructing (e.g., of the upper airway causing airway compromise or of the GI tract necessitating parenteral feeding), edema that is obstructing (e.g., of the lower GI tract), extradermatologic manifestations (e.g., pulmonary nodules, encephalitis, myopericarditis, or ocular infections), and sepsis. Detailed characteristics of severe disease are available at https://www.cdc.gov/poxvirus/monkeypox/clinicians/treatment.html#anchor_1655488137245. ** Persons with underlying medical conditions (e.g., severe or moderate immunocompromise [https://www.cdc.gov/poxvirus/monkeypox/clinicians/people-with-HIV.html]); bacterial superinfections; or complications, including strictures, edema, and infections of the penile foreskin, vulva, urethral meatus, or anorectum, which could require procedural intervention (e.g., urethral catheterization, colostomy, or surgical debridement). This also includes those with or at risk for ocular lesions (i.e., presence of eyelid lesions, facial lesions near the eyes, or finger or hand lesions in patients unable to avoid touching their eyes [for whom autoinoculation is a concern]). Detailed characteristics of persons at risk for severe disease are available at https://www.cdc.gov/poxvirus/monkeypox/clinicians/treatment.html#anchor_1655488137245. ^††^ https://www.cdc.gov/poxvirus/monkeypox/clinicians/case-definition.html ^§§^ This figure is a comprehensive synthesis of heterogeneous evidence and is intended to foster strategic decision-making rather than serve as a prescriptive treatment guideline.

**TABLE:** Summarized mechanisms of action, administration recommendations, adverse events, clinical considerations, and supporting data about medical countermeasures that can be used to treat mpox* — United States, January 2023

Consideration	Medical countermeasures				
	Tecovirimat (Tpoxx or ST-246)	Brincidofovir (Tembexa or CMX001)	Cidofovir (Vistide)	VIGIV	Trifluridine (Viroptic)
**Description**	An OPXV-specific antiviral with limited activity against unrelated RNA or DNA viruses	Lipid-conjugated analog of cidofovir with different properties compared with cidofovir	Monophosphate nucleotide analog used to treat viral infections including cytomegalovirus	Solvent- or detergent-treated, filtered sterile solution of purified immune globulin from human plasma of persons with antibodies to vaccinia virus	Nucleoside analog used to treat ocular viral infections including herpes simplex virus keratitis
**Mechanism of action against OPXVs**	Inhibits association of VP37 (a protein encoded by and highly conserved across the OPXV genus) with a cellular protein, preventing formation of egress-competent envelope virions necessary for cell-to-cell dissemination of virus	Once inside cells, the lipid ester linkage is cleaved to form cidofovir, which is then converted to CDP, which selectively inhibits OPXV DNA polymerase.	Intracellularly converted to CDP which selectively inhibits OPXV DNA polymerase	Provides passive antibody which might have cross-reactivity across the OPXV genus	Thymidine analog that interferes with DNA synthesis in cultured mammalian cells with selective toxicity to viral replication of OPXVs
**Dose**	600 mg^†^	200 mg^§^	5 mg/kg	6,000–9,000 units/kg^¶^	One drop into affected eyes
**Route**	Oral (capsules) or IV**	Oral (tablets or suspension)	IV	IV	Topical
**Frequency**	Twice daily (40 kg to <120 kg) Three times daily (>120 kg)	Once weekly	Once weekly	Single dose but can be repeated depending on duration of illness and severity of immunocompromise	Every 2 hrs when awake for the first 2 wks (maximum nine drops per eye per day) Then, four times daily for an additional 2 wks
**Duration**	2 wks unless indication to prolong^††^	2 wks unless indication to prolong^††^	2 wks unless indication to prolong^††^	NA	>4 wks
**Potential adverse events**	Headache, nausea, diarrhea, itching, and abdominal pain; labeled contraindication for IV administration when creatinine clearance is <30 mL/min^§§^	Abdominal pain, diarrhea, nausea, vomiting, and elevated liver enzymes	Nephrotoxicity, nausea, vomiting, and acidosis	Adverse events associated with infusion of immunoglobulins (e.g., headache, diaphoresis, erythema, anaphylaxis, thrombosis, acute kidney injury, and volume overload)	Adverse events associated with topical use (e.g., burning, stinging, or eyelid edema)
**Warnings**	Other warnings: Clearance of hydroxypropyl-β-cyclodextrin is dependent on glomerular filtration, and caution is advised in patients with mild to moderate renal impairment, and in pediatric patients <2 yrs.	BBW: Increased mortality seen in a 24-wk placebo-controlled trial for CMV prophylaxis in hematopoietic stem-cell transplant recipients. Prolonging therapy beyond 2 wks should be considered with caution, and currently requires FDA authorization on an individual patient basis through an emergency IND request. Other warnings: Neutropenia Potential human carcinogen, teratogen, and causing hypospermia	BBW: Severe nephrotoxicity resulting in dialysis or contributing to death. IV prehydration and administration of probenecid must be used with each infusion. Neutropenia Potential human carcinogen, teratogen, and causing hypospermia Other warnings: Decreased intraocular pressure, metabolic acidosis	Other warnings: Hypersensitivity, renal dysfunction, interference with blood glucose testing, thrombotic events, aseptic meningitis syndrome, hemolysis, transfusion-related acute lung injury, and transmission of infectious agents from human plasma	Other warnings: Continuous administration beyond the recommended duration might cause corneal epithelial toxicity.
**Drug interactions**	Might reduce levels of NNRTI rilpivirine Might increase concentration of blood glucose-lowering agent repaglinide Decrease concentration of midazolam	Protease inhibitors, cobicistat, and fostemsavir might increase brincidofovir concentration.	Cidofovir has limited interactions; however, it necessitates coadministration with probenecid which has numerous interactions (e.g., zidovudine, beta-lactam antimicrobials, diuretics, NSAIDs, and ACEi) that need to be monitored.	Vaccination with live virus vaccines (e.g., varicella measles, mumps, and rubella) should be deferred for 3 mos after use.	NA
**Data gleaned from selected animal studies** ^¶¶^	Cynomolgus macaques were lethally challenged IV with MPXV and treated on day 4, 5, and 6 postchallenge. Treatment with tecovirimat for 14 days resulted in statistically significant improvement in survival relative to placebo, except when given starting at day 6 postchallenge.	In a lethal rabbitpox and mousepox (ectromelia) model of infection, treatment with brincidofovir resulted in statistically significant improvement in survival relative to placebo, except when given starting at day 6 postchallenge in the mousepox study.	In a lethal dormouse model of MPXV, cidofovir-treated mice reduced mortality from 100% to 19%.	In a mouse-tail lesion model, VIGIV exerted a protective effect against vaccinia infection when compared with a negative control.	In a study of 56 rabbits with vaccinia keratitis, trifluridine significantly reduced clinical disease and viral culture positivity.
**Data gleaned from use in humans before and during the 2022 outbreak**	Of 255 patients treated during the current outbreak,*** the median interval to first subjective improvement was 3 days with limited adverse events reported.	Limited use as monotherapy; used (unsuccessfully) alternating with cidofovir for severe cowpox infection in a kidney transplant recipient	Limited use as a sole agent; used for ocular cowpox infection with unclear benefit and once (unsuccessfully) alternating with brincidofovir for severe cowpox infection in a transplant recipient	Evidence for smallpox prevention when given to high-risk contacts Mixed evidence for efficacy for treatment of progressive vaccinia	Used successfully off-label for ocular complications of vaccinia virus vaccination
**Pregnancy, breastfeeding, and fertility considerations**	Likely safe in pregnancy and breastfeeding without affecting fertility	Not recommended for pregnancy or breastfeeding Might cause irreversible infertility in males	Not recommended for pregnancy or breastfeeding Might cause irreversible infertility in males	Likely safe in pregnancy and breastfeeding without affecting fertility	Negligible systemic absorption when administered into the eye; likely safe in pregnancy and breastfeeding without affecting fertility
**CNS considerations** ^†††^	Penetrates well	Penetrates to uncertain degree	Penetrates to limited degree	Penetrates to limited degree	NA
**Resistance considerations**	Single point mutation can confer resistance.^§§§^	Single point mutation can confer minor resistance; however, multiple mutations are needed for high-level resistance and the resultant virus becomes less virulent.	Single point mutation can confer minor resistance; however, multiple mutations are needed for high-level resistance and the resultant virus becomes less virulent.	NA	Although in vitro resistance has not been reported, the possibility of resistance exists.
**Miscellaneous considerations**	High-fat (approximately 600 calories and 25g fat) meal required with each oral dose IV administration should be considered in those who are unable to take oral therapy, unable to consume high-fat meal, have impaired gastrointestinal absorption (e.g., gastric bypass, diarrhea, or evidence of other gastrointestinal disfunction that might negatively affect drug absorption), or fail to improve on approximately 7 days of oral therapy.	Must be taken on an empty stomach or with a low-fat meal (approximately 400 calories with 25% of calories from fat) Might have superior antiviral activity to that of cidofovir because of increased cellular uptake Should not be administered within 1 wk of cidofovir because both form the same active metabolite (CDP), which has a prolonged duration of action	Must be given with probenecid to minimize nephrotoxicity Probenecid might have substantial drug interactions and consultation with a pharmacist is advised. Should not be administered within 1 wk of brincidofovir because both form the same active metabolite (CDP), which has a prolonged duration of action	Vaccinia-specific antibody, which might cross-react with MPXV Might interfere with endogenous antibody production and IgG antibody testing When used to treat vaccinia keratitis in rabbits, VIGIV resulted in prolonged scarring and stromal edema.	Can cause permanent limbal stem cell deficiency with prolonged use

**Abbreviations**: ACEi = angiotensin converting enzyme inhibitor; BBW = black box warning; CDP = cidofovir diphosphate; CMV = cytomegalovirus; CNS = central nervous system, EA-IND = expanded access - investigational new drug; FDA = Food and Drug Administration; IgG = immunoglobulin G; IND = investigational new drug; IV = intravenous; MPXV = *Monkeypox virus*; NA = not applicable; NNRTI = non-nucleoside reverse transcriptase inhibitor; NSAIDs = nonsteroidal anti-inflammatory drugs; OPXV = orthopoxvirus; VIGIV = vaccinia immune globulin intravenous.

* This table is a comprehensive synthesis of the heterogeneous evidence and is intended to foster strategic decision-making rather than serve as a prescriptive treatment guideline.

^†^ Dosing for persons <40 kg and children is available at https://www.cdc.gov/poxvirus/monkeypox/pdf/tecovirimat-ind-protocol-cdc-irb.pdf. ^§ ^Dosing for persons <48 kg and children is available at https://www.accessdata.fda.gov/drugsatfda_docs/label/2021/214460s000,214461s000lbl.pdf.

^¶^ Data suggest that an aggressive early dosing regimen for patients with severe immunocompromise might be most beneficial; for this reason, a dose in the higher range (9,000 units/kg) early in the clinical course, potentially followed by an additional dose 3–4 days later, might help saturate viral antigens and halt viremia and viral replication.

** IV administration requires a syringe pump, a device that might not be easily accessible in all health care settings and has a slow infusion longer than 6 hours, irrespective of total infusion volume.

^††^ Per EA-IND, the standard duration of tecovirimat treatment is 14 days, with clinical data being limited to a 14-day course. Based on individual patient risk-benefit assessment and disease progression, tecovirimat and cidofovir may be extended beyond 14 days, or shortened because of lack of virologic or clinical response or adverse event occurrence. Extension of brincidofovir currently requires FDA authorization on an individual patient basis through an emergency IND request secondary to concerns related to the BBW stated within the table.

^§§^ Secondary to potential accumulating of hydroxypropyl-β-cyclodextrin, an excipient in the IV tecovirimat formulation, which is eliminated through glomerular filtration. IV tecovirimat may be used with caution in patients with renal impairment (creatine clearance <30 mL/min) only if drug absorption via enteral administration is not anticipated to be dependable or feasible, and based on the risk-benefit assessment by the treating clinician that determines IV tecovirimat clinically necessary.

^¶¶^ Other animal studies examine the use of these agents; the studies noted within the table were used to gain FDA approval or provide evidence of efficacy for its off-label use.

*** https://www.cdc.gov/mmwr/volumes/71/wr/mm7137e1.htm?s_cid=mm7137e1_w

^††† ^CNS penetration was determined based on available animal and human data. Well: data suggested that an MCM was able to cross the blood-brain barrier and enter intrathecal space at concentrations known to be effective; Uncertain: data suggested that an MCM can cross the blood-brain barrier, but unclear if at sufficient concentrations to be effective; Limited: evidence suggested that an MCM does not or would not cross the blood-brain barrier. The theoretical utility of any medical countermeasure in clearing MPXV from the central nervous system is unknown.

^§§§ ^To date, <0.5% specimens (out of >5,000 specimens) sent to CDC for testing have been found to develop resistance within the current outbreak.

**Tecovirimat. **Tecovirimat is administered two to three times daily (depending upon patient’s weight), typically for 2 weeks. Based on the favorable safety and efficacy profile of tecovirimat compared with other MCMs, if only one MCM is administered, it should be tecovirimat, unless there is a contraindication such as a previous adverse event after receiving the drug. The pharmacokinetics of orally administered tecovirimat taken with a fatty meal compare favorably with those of IV tecovirimat. IV tecovirimat (which is currently available in limited supply) should be prioritized for patients who are unable to take oral medications or fatty meals with each dose, have gastrointestinal disease that might impair absorption (e.g., new or chronic diarrhea), or have diffuse and disseminated infection.^††††^ For patients for whom IV tecovirimat is indicated, prepositioned oral tecovirimat should be administered until the IV formulation is obtained.

Patients with severe immunocompromise might benefit from extended treatment (i.e., >14 days) if new confirmed OPXV lesions occur or existing lesions worsen despite treatment. Data from animal studies suggest it might be safe to extend tecovirimat treatment (*18*). Clinicians should carefully consider the risks and benefits of extending treatment, and extensions of short, defined intervals should be used (e.g., an additional 3–7 days) with close monitoring for safety signals and clinical response. Tecovirimat resistance has been detected in a small number of patients with advanced HIV who received tecovirimat for periods of weeks to months (*19*). Resistance can also develop as the result of subtherapeutic levels of tecovirimat (e.g., because of medication noncompliance or because fatty meals are not taken with the oral formulation). Testing for tecovirimat resistance and pharmacokinetics^§§§§^ for public health surveillance purposes is encouraged when any new lesions form after ≥7 days of treatment.

**Brincidofovir and cidofovir.** One of these drugs can be added to tecovirimat treatment for patients with (or at risk for) severe mpox. They are usually administered once weekly for 2 weeks. One animal study suggests that combined treatment (tecovirimat and brincidofovir, the prodrug of cidofovir) might have synergistic efficacy (*20*). Brincidofovir or cidofovir without tecovirimat should typically only be administered to patients in whom tecovirimat is contraindicated. Brincidofovir and cidofovir should not be used simultaneously or within 1 week of one another, because they form the same active metabolite (cidofovir diphosphate), which has a prolonged duration of action. Both drugs have FDA black box warnings and other safety considerations that require close monitoring. Diarrhea has been commonly reported in patients who receive brincidofovir^¶¶¶¶^; diarrhea of any etiology might impair absorption of orally administered tecovirimat and indicate a need for IV tecovirimat. In vitro studies suggest that brincidofovir might have superior antiviral activity to that of cidofovir against variola virus, likely because of better cellular uptake (*21*,*22*); however, because data are limited, side effect profiles should be prioritized when choosing between the two drugs. Development of resistance to brincidofovir or cidofovir is less likely to occur than is resistance to tecovirimat (*23*,*24*).

**VIGIV.** VIGIV administered as a single dose provides passive immunoglobulin (Ig) G antibodies against vaccinia virus, which might provide some cross-protection across the OPXV genus, including for MPXV. During the current outbreak, it has been recommended for mpox patients unable to mount a sufficiently robust immune response to clear the virus (e.g., because of HIV-related CD4 count <350 or after solid organ transplantation). Although its effectiveness for mpox is unknown, the safety profile is believed to be favorable; however, caution should be exercised when administering VIGIV to patients with ocular mpox involving the cornea because of a report of an animal study of vaccinia keratitis in which VIGIV was associated with persistent corneal scarring (*25*,*26*). VIGIV is available in limited supply. Subsequent dosing (i.e., redosing) decisions should be made on a case-by-case basis in consultation with CDC. Clinical characteristics and laboratory results that might trigger consideration of additional doses of VIGIV include mpox lesions affecting a large percentage of a patient’s body surface at the time of diagnosis, emergence of new mpox lesions (or expanding borders on existing lesions) several days after VIGIV, persistent severe immunocompromise (e.g., as evidenced by low CD4 values and undetectable OPXV IgM despite attempts to optimize immune function), lesions affecting mobility or that are concerning for long-term sequelae such as sexual dysfunction, and inability to maximally use other MCMs because of adverse events or contraindications.

**Trifluridine.** Trifluridine is an ophthalmic antiviral drug that has been shown to inhibit replication of several viruses, including vaccinia virus (*27*) and has demonstrated efficacy against ocular vaccinia virus infections in animal models (*25*,*28*) and humans (*28*,*29*). Continuous administration beyond the recommended 4-week duration of treatment should be avoided because of the risk for corneal epithelial toxicity (*30*).

## Considerations in the Management of Severe Mpox Cases

Severe mpox (including ocular infections, neurologic complications, myopericarditis, mucosal lesion complications, and uncontrolled viral spread) have been reported. Manifestations of these complications, recommended MCMs, and other clinical considerations for each type of infection (e.g., involvement of specific clinical subspecialists) are summarized (Box).

BOX Important clinical considerations for management of severe mpox* — United States, January 2023

**Ocular infections**

- **Clinical manifestations:** Symptoms include eye pain, redness, drainage, foreign body sensation, vision changes or loss, or periorbital swelling. Involvement of the ocular surface can manifest as blepharitis, conjunctivitis, or keratitis; discrete lesions might be present. Lesions can also occur on external areas including the eyelids.
- **Diagnostic findings:** In a patient with known or suspected mpox, ocular infection can be confirmed by testing swabs from periorbital, lid or intraocular lesions for OPXV by PCR.
- **Treatment:** Prompt initiation of tecovirimat and topical administration of trifluridine should be considered. Trifluridine can also be used prophylactically in patients with mpox who are at high risk of ocular infection (e.g., lesions near the eye). Other systemic MCMs should be considered on a case-by-case basis. Lubricants and topical antibiotics may be considered for symptomatic management and prevention of complications.
- **Other considerations:** Obtain ophthalmology consultation.^†^ Adverse events might occur from prolonged use of trifluridine. In addition, one animal study suggests increased risk of corneal scarring when VIGIV is administered in the setting of OPXV keratitis. Extensive use of agents that can further irritate the eye, such as topical povidone-iodine, might be avoided. Appropriate measures to prevent, diagnose, and treat ocular coinfections and superinfections should be taken.

**Neurologic complications**

- **Clinical manifestations:** Encephalitis and myelitis can occur. Severe headache, back or neck pain, altered mental status, seizures, or focal neurologic deficits in a patient with mpox or recently recovered from mpox should prompt suspicion for neurologic complications.
- **Diagnostic findings:** CSF might demonstrate a lymphocytic-predominant pleocytosis with protein elevation and normal glucose; availability of mpox-specific CSF testing is limited and consultation with CDC is suggested. MRI might show lesions in the brain or spinal cord which might or might not enhance.
- **Treatment:** Treatment of mpox-associated neurologic disease should involve MCMs and might involve immunomodulatory or immunosuppressive therapy (e.g., steroids, intravenous immunoglobulin, or plasmapheresis or plasma exchange). Clinicians treating mpox-associated neurologic disease should weigh the risks and benefits of immunosuppressive agents when direct viral neuroinvasion is a possibility. Data suggest tecovirimat penetrates the CNS well; although brincidofovir, cidofovir, and VIGIV might penetrate the CNS, the extent is either uncertain (brincidofovir) or limited (cidofovir and VIGIV).
- **Other considerations:** Consider neurology consultation. Neurologic disease related to mpox might be because of direct viral invasion of the CNS or resultant autoimmune disease from antigenic stimulus. Other neurologic diseases with similar presentations should be investigated (e.g., infectious diseases such as viral encephalitides and syphilis, and autoimmune, parainfectious, and vascular conditions).

**Myopericarditis**

- **Clinical manifestations:** New complaints of chest pain, shortness of breath, or palpitations in a patient with ongoing or recent mpox should prompt consideration of myopericarditis.
- **Diagnostic findings:** Similar findings to those associated with myopericarditis from etiologies other than mpox might be observed, including elevations in cardiac biomarkers, changes in electrocardiogram and on cardiovascular MRI, and pathologic changes of the myocardium.
- **Treatment:** Standard of care for myopericarditis should be considered; MCMs might also play a role by limiting viral spread to myocytes or decreasing the production of viral antigens responsible for the inflammatory response.
- **Other considerations:** Consider cardiology consultation. Other causes of myopericarditis should be investigated, including other viral infections or recent receipt of a vaccination that can be associated with myopericarditis.
**Complications associated with some mucosal (oral, rectal, genital, and urethral) lesions**
- **Clinical manifestations:** Symptoms can include impaired activities of daily living (e.g., feeding, urination, or defecation) from painful or obstructing rectal, urinary tract, oral, and genital lesions, especially if associated with strictures, substantial edema, severe lymphadenopathy, or necrosis. Lesions might expose deep tissue including muscle or bone, and myonecrosis can occur. Healing might be slow, and scarring can result in strictures.
- **Diagnostic findings:** Complications associated with mucosal lesions can be diagnosed by physical examination in conjunction with other diagnostic testing; the diagnosis of mpox can be made by sampling mucosal or other lesions.
- **Treatment:** Prompt initiation of systemic MCMs should be considered. Some patients have required intubation, urinary catheterization, or placement of enteric tubes. Early and aggressive treatment might prevent such complications. Routine use of topical antimicrobial agents, particularly over-the-counter options, is not indicated and might cause irritation, contact dermatitis, or delayed wound healing.^§ ^Debridement is generally not recommended.
- **Other considerations:** Specialists (e.g., surgery, urology, or gastroenterology) should be consulted early in the clinical course. Symptomatic management, and especially pain control, is an important component of treatment. Coinfections and superinfections should be diagnosed and treated promptly. Sequelae are not fully known but can result in substantial morbidity (e.g., scarring leading to functional impairment, or necrosis necessitating surgical debridement, penectomy, or amputation of extremities).

**Complications from uncontrolled viral spread in moderately to severely immunocompromised patients**

- **Clinical manifestations**: Numerous, large, coalescing, or necrotic lesions of the skin can occur in patients with severe immunocompromise. Other organ systems (e.g., gastrointestinal tract, liver, lungs, brain, or adrenal glands) can be involved, resulting in signs and symptoms of organ dysfunction irrespective of severity of cutaneous lesions. Overwhelming systemic illness including sepsis can occur and might progress to death.
- **Diagnostic findings**: Uncontrolled viral spread can manifest as the appearance of new skin lesions or worsening of existing lesions. Involvement of other organ systems can result in a range of findings on physical exam and laboratory investigations (e.g., gastrointestinal obstruction, severe pneumonia, empyema, encephalitis, intractable hypotension, or transaminitis). Alternate or coinciding causes of severe illness should be investigated.
- **Treatment:** Immune function should be optimized through interventions such as effective HIV antiretrovirals and reduced immunomodulatory therapy as feasible. Prompt initiation of tecovirimat (potentially the intravenous formulation), and possible combination with either cidofovir or brincidofovir, and VIGIV, should be considered. Wound care is critical to ensure healing and prevent superinfection and autoinoculation.^§^ Diffuse skin lesions might cause insensible fluid losses requiring intensive fluid management.
- **Other considerations**: Consider consultation with experts in infectious diseases, critical care, dermatology, wound care, gastroenterology, and surgery (e.g., general surgery, plastic surgery, and burn experts) as indicated. Administration of MCMs for extended durations (>14 days) might be reasonable if clinically indicated (e.g., new or progressive mpox lesions occur). The role of immune dysregulation in severe mpox illness is not known; there is no high-quality evidence to support or refute the use of steroids and other immunomodulators, and clinicians should weigh the risks and benefits of such therapies because optimal immune function aids recovery from mpox. Supportive care and close clinical monitoring for occurrence of complications such as secondary bacterial infections and sepsis can be critical in patients with severe mpox illness.

**Abbreviations**: CNS = central nervous system; CSF = cerebrospinal fluid; MCM = medical countermeasure; MRI = magnetic resonance imaging; OPXV = orthopoxvirus; PCR = polymerase chain reaction; VIGIV = vaccinia immune globulin intravenous.

* This report is a comprehensive synthesis of the heterogeneous evidence and is intended to foster strategic decision-making rather than serve as a prescriptive treatment guideline.

^†^ Urgent ophthalmology consultation and management is particularly important for patients with eye pain, vision loss, or worsening ocular symptoms.

^§ ^https://www.aad.org/public/diseases/a-z/monkeypox-self-care

**Ocular infections. **These can manifest as blepharitis, conjunctivitis, conjunctival lesions, keratitis, and vision loss. Ocular infections have occurred when MPXV infects the eye or periorbital area, usually via autoinoculation or local spread from nearby lesions (*31*).

**Neurologic complications.** Encephalitis and myelitis have been reported in some mpox patients (*32*). Whether these result from direct viral invasion of the central nervous system or autoimmune disease from antigenic stimulus is not known. Cases involving neurologic complications have rarely been reported to CDC, but have occurred in immunocompetent persons, despite resolving mpox skin lesions.

**Myopericarditis.** Myopericarditis cases have been reported among immunocompetent patients (*1*,*32*,*33*). The pathogenic mechanism is uncertain but might result from direct viral invasion or lymphocytic infiltration of the myocardium or pericardium, its sequelae (e.g., myonecrosis or myocardial fibrosis), or an autoimmune process.

**Complications associated with some mucosal (oral, rectal, genital, and urethral) lesions. **Certain mucosal surface lesions can cause strictures and other complications that impair activities of daily living (e.g., reduced oral intake, painful bowel movements, impaired urination, and airway obstruction).

**Complications from uncontrolled viral spread in immunocompromised patients.** Moderately or severely immunocompromised patients (e.g., advanced HIV and organ transplant recipients) have sometimes developed diffuse and disseminated lesions that have involved multiple organ systems, possibly because of persistent viremia or uncontrolled viral growth (*12*). Overwhelming systemic illness has resulted in death.

## Other Clinical Considerations

**Immune dysregulation. **Earliest optimization of immune function is critical to favorable outcomes. Mpox patients with advanced HIV who have recently started ARVs and who then experience persistent or progressive lesions might manifest features commonly associated with immune reconstitution inflammatory syndrome; whether immune dysregulation is the cause of illness or the immune system is unable to effectively clear MPXV from infected cells is not known. Until definitive data are available, administration of systemic immunomodulators (including steroids) to patients with mpox should be undertaken with caution; models suggest that steroids are associated with severe illness and even death in OPXV-infected animals (*34*).

**Lesions that persistently test positive. **Positive OPXV and MPXV polymerase chain reaction (PCR) test results are expected until lesions resolve; therefore, serial testing of lesion specimens is not informative unless new lesions appear or lesions progress despite tecovirimat treatment. Test results do not guide duration of infection control policies because patients are considered infectious until all lesions have scabbed, the scabs have fallen off, and healthy tissue is visible underneath.

**Prolonged occurrence of new lesions despite appropriate MCMs*. ***If progressive lesions are noted, particularly after reversal of immunocompromise, diagnostic evaluation should include testing new lesions for OPXV and other infections, and evaluation for superinfections, noninfectious processes such as erythema multiforme, and immunologic function. Laboratories, such as CDC’s poxvirus laboratory,***** can test for presence of viable virus from lesion specimens using culture techniques that might guide patient care. If viral culture is unavailable, evaluating trends in PCR cycle threshold values might be informative.

## Knowledge Gaps and Next Steps

Knowledge gaps regarding optimal treatment of severe mpox are best addressed through data collected during randomized controlled trials and other carefully controlled research studies. Patients enrolled in well-designed studies might benefit from the close monitoring provided by these studies (e.g., effective adjustments of tecovirimat doses are made as part of the STOMP trial based on serially monitored pharmacokinetic parameters). Understanding the role of immune dysregulation in the clinical course of severely immunocompromised HIV patients started on ARVs was frequently recognized as a knowledge gap: CDC has partnered with NIH to study this (Virologic and Immunologic Characteristics of Severe Mpox Among Persons with Advanced HIV [VIRISMAP] study).^†††††^ Clinicians and health departments are encouraged to contact CDC when treating mpox in a patient recently started on ARVs. Controlled studies focused on understanding the impact of monotherapy or combination therapy on virus shedding, duration of illness, and clinical outcomes are needed, particularly for patients with severe immunocompromise. Public health laboratories, academic laboratories, and CDC continue to sequence the F13L gene (the tecovirimat target) to assess F13L viral mutations that might be associated with resistance. Phenotypic testing to evaluate resistance is also occurring at CDC.^§§§§§^ Analysis of anti-OPXV antibody levels and viral neutralization antibody levels are ongoing at CDC and are needed to develop guidance about redosing of VIGIV. Laboratories should consider examining T-cell and humoral responses to mpox in immunocompromised patients because immune response is crucial to viral clearance, and this data might facilitate development of improved clinical guidance (*35*).

Until data from controlled studies are available, observational data from patients treated under IND might provide insights into clinical outcomes. Providers administering MCMs under IND programs should complete and submit optional data collection forms to facilitate improved understanding of the role of MCMs. CDC will update guidance, as appropriate, as new data emerge.

**Summary**

**What is already known about this topic?**

During the 2022 global monkeypox (mpox) outbreak, some patients have experienced severe clinical manifestations. Medical countermeasures (MCMs) developed to treat smallpox have been used to treat mpox.

**What is added by this report?**

Data relevant to the use of tecovirimat, brincidofovir, cidofovir, trifluridine ophthalmic solution, and vaccinia immune globulin intravenous were reviewed. Animal models, MCM use for human cases of related orthopoxviruses, unpublished data, input from clinician experts, and experience during CDC mpox consultations were also evaluated to develop interim clinical treatment considerations.

**What are the implications for public health practice?**

Until data from controlled studies are available, these interim clinical considerations facilitate strategic decision-making about the use of MCMs to manage specific severe manifestations of mpox.

## Footnotes

* https://www.cdc.gov/poxvirus/monkeypox/index.html

†
https://www.cdc.gov/poxvirus/monkeypox/clinicians/pain-management.html

§ CDC offered an mpox clinical consultation service for the ongoing mpox outbreak. The existence of this service was widely publicized during CDC Clinician Outreach and Communication Activity calls, CDC’s Health Alert Network messages, national conference presentations, and other communications. Health care providers seeking clinical consultations can continue to contact CDC mpox experts through the CDC Emergency Operations (770-488-7100) or by email (poxvirus@cdc.gov).

¶
https://emergency.cdc.gov/han/2022/han00475.asp

**
https://aspr.hhs.gov/phemce/Pages/goals.aspx

††
https://www.cdc.gov/poxvirus/monkeypox/about/science-behind-transmission.html

§§
http://web.archive.org/web/20060117201021/http://www.fda.gov/cber/label/smalwye102502LB.pdf

¶¶ https://www.fda.gov/files/vaccines%2C%20blood%20%26%20biologics/published/Package-Insert---ACAM2000.pdf

***
https://www.cdc.gov/poxvirus/monkeypox/clinicians/pain-management.html

†††
https://emergency.cdc.gov/han/2022/han00481.asp

§§§
https://www.stomptpoxx.org/main

¶¶¶
https://www.cdc.gov/poxvirus/monkeypox/clinicians/people-with-HIV.html

**** https://www.cdc.gov/poxvirus/monkeypox/clinicians/pediatric.html; https://www.cdc.gov/poxvirus/monkeypox/clinicians/pregnancy.html

††††
https://www.cdc.gov/poxvirus/monkeypox/clinicians/obtaining-tecovirimat.html

§§§§
https://www.cdc.gov/poxvirus/monkeypox/pdf/attachment-5-optional-pk-sample-testing.pdf

¶¶¶¶
https://www.accessdata.fda.gov/drugsatfda_docs/label/2021/214460s000,214461s000lbl.pdf

***** CDC’s poxvirus laboratory can be reached at poxvirus@cdc.gov and poxviruslab@cdc.gov.

††††† https://www.cdc.gov/poxvirus/mpox/clinicians/treatment.html

§§§§§
https://www.cdc.gov/poxvirus/monkeypox/pdf/Optional-Lesion-Sample-to-CDC-for-Resistance-Testing.pdf
